# Comparison and analysis of the clinicopathological features of SCEO and ECOM

**DOI:** 10.1186/s13048-019-0485-5

**Published:** 2019-01-30

**Authors:** Ting Wang, Xiaodan Zhang, Zhiying Lu, Junyan Wang, Keqin Hua

**Affiliations:** Department of Gynecology, obstetrics and gynecology hospital, Fudan university, Shanghai, 200,090 China

**Keywords:** Endometrial carcinoma, Ovarian cancer, Synchronous primary cancer of the endometrium and ovary (SCEO), Endometrial cancer with ovarian metastasis (ECOM)

## Abstract

**Objective:**

The aim of our study was to evaluate and compare the differences in the clinicopathological variables and overall survival (OS) of synchronous primary cancers of the endometrium and ovary (SCEO) and endometrial cancer with ovarian metastasis (ECOM). In addition, we aimed to determine the characteristics of and effective treatments for patients with SCEO to avoid misdiagnosis and overtreatment.

**Materials and methods:**

A review of medical records from January 2009 to January 2017 revealed 111 patients with coexisting ovarian and endometrial carcinoma diagnosed at the Obstetrics and Gynecology Hospital of Fudan University. Clinicopathological variables were analysed using the Chi square test and Student’s t test. The survival rate was estimated using the Kaplan-Meier method, and statistical significance was analysed using the logarithmic rank test (univariate analysis).

**Results:**

There were 51 cases of SCEO and 60 cases of ECOM. The mean age at diagnosis was 53.96 years and 55.41 years, respectively. There were no differences in age, menopausal status, BMI, CA125 level or complaints between the two groups. The 5-year survival rates were 58.8 and 36.7%, respectively (*P* < 0.001). Significant differences were found in the endometrial tumour classification, ovarian cancer stage, and lymph node and omentum metastasis between SCEO and ECOM.

**Conclusions:**

The differences found between SCEO and ECOM are of great clinical significance. Our results reveal useful prognostic and clinicopathological features. More aggressive therapies should be administered to both SCEO and ECOM patients, especially elderly patients and those with menopause, endometrial tumours, advanced omentum metastasis, and lymph node dissection.

## Introduction

With clinical experience and the progression of scientific research, the idea that two or more cancers can occur at the same time in the female reproductive system is becoming more accepted. The most common type is synchronous primary cancers of the endometrium and ovary (SCEO). Although the incidence of SCEO is limited, it is easily confused with endometrial cancer with ovarian metastasis (ECOM), as well as stage III endometrial cancer. SCEO is found in 10% of patients with ovarian cancer and 5% of patients with endometrial cancer [1]. The diagnosis of both conditions significantly affects the prognosis of the endometrium; thus, the distinction between SCEO and ECOM is very important. Histopathological criteria have been used for this purpose. Ulbright and Roth et al. [2] established the histological criteria of SCEO and ECOM in 1985, and Scully et al. [3] subsequently provided a more detailed list of features for distinguishing SCEO from ECOM. The aim of the study was to compare the clinicopathological variables and prognostic factors of SCEO and ECOM.

## Materials and methods

Between January 2009 and January 2017, 111 patients with coexisting of ovarian and endometrial carcinoma were diagnosed at the Obstetrics and Gynecology Hospital of Fudan University; 51 patients had SCEO, and 60 patients had ECOM. Patients with neoadjuvant chemotherapy and borderline tumours were excluded.

The collected clinical data included age, BMI, menopausal state, symptoms, CA125 serum level, and follow-up treatments, as well as histopathological and surgical details, such as the grade, myometrial invasion depth, and lymphatic vascular space invasion (LVSI).

All patients’ stages were reviewed and updated. These stages were based on the endometrial and ovarian cancer staging criteria set by the International Federation of Gynecology and Obstetrics (FIGO). The Ulbright and Roth criteria and the Scully criteria were used to differentiate synchronous tumours from metastatic tumours as follows: (1) no direct connection between two cancers; (2) no myometrial infiltration or superficial myometrial infiltration; (3) no lymphatic and intravascular infiltration; (4) tumours mainly present in the ovaries and endometrium; (5) often limited to primary or only minor metastases; (6) often accompanied by endometrial atypical hyperplasia; (7) sometimes accompanied by ovarian endometriosis; and (8) same or different histological types. The first 5 items were used as the main diagnostic criteria. The diagnosis of ECOM required either the presence of a multinodular ovarian pattern (major criterion) or 2 or more minor criteria, such as (1) small-diameter ovarian tumours, < 5 cm; (2) bilateral ovarian invasion and multiple nodular ovarian lesions; (3) deep myometrial infiltration; (4) tumour-infiltrating vessels; and (5) oviduct violation with conformation of the clinical symptoms and pathological results with more than 2 of the above criteria.

SPSS version 16 (SPSS, Chicago, IL) was used for the statistical analysis. Clinical pathological variables, including categorical data, were analysed using the Chi square test, and continuous data were analysed by the Student t test. The survival rate was estimated by the Kaplan-Meier method, with statistical significance set at 0.05 and determined using the log-rank test (univariate analysis). The actuarial curves were compared using the log-rank test, with statistical significance set at 0.05. A probability value of less than 0.05 was considered statistically significant.

## Results

**Fig. 1:**
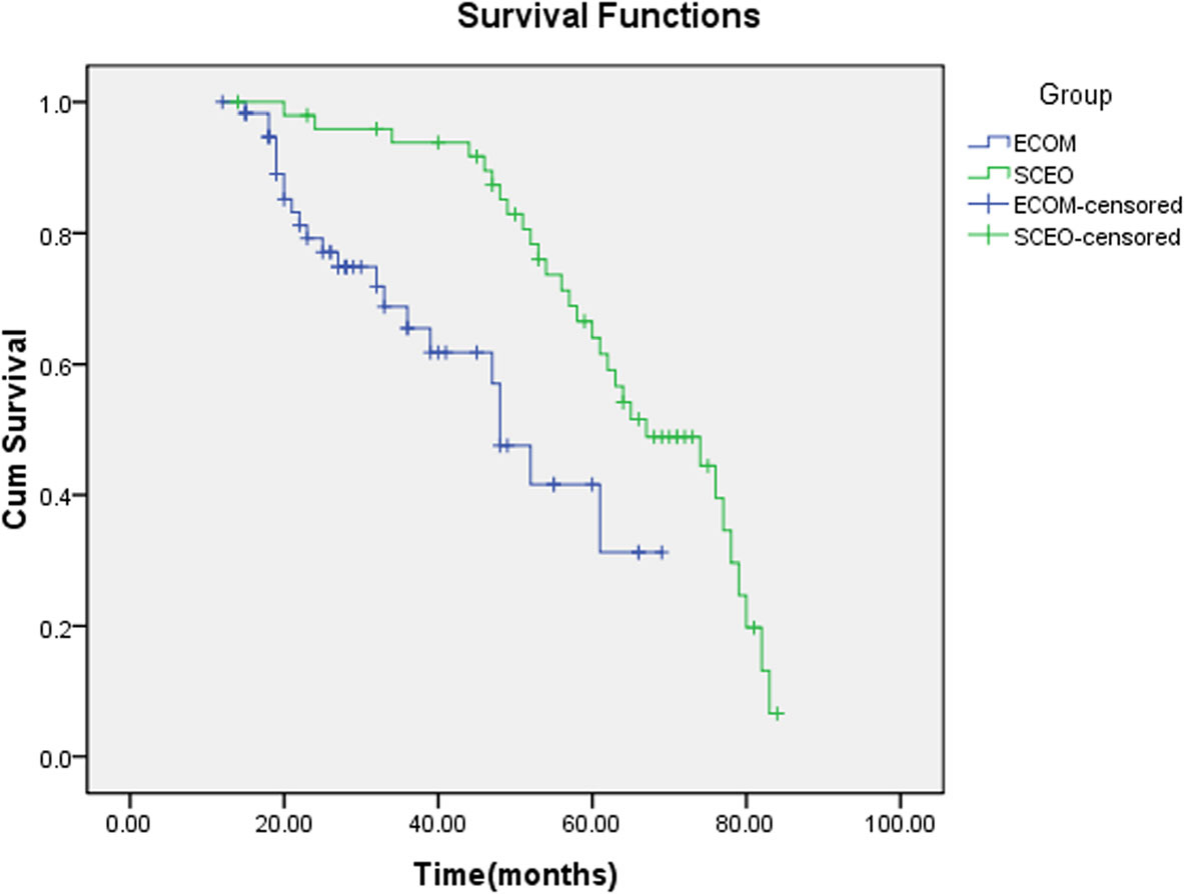
Survival analysis of patients with ECOM and SCEO

In all, 51 cases of SCEO and 60 cases of ECOM were diagnosed, as shown in Table 1. In the SCEO and ECOM groups, there were 40 and 43 cases of the same pathological type, and 11 cases and 17 cases with different pathological types, including serous adenocarcinoma, clear cell carcinoma, and mixed carcinoma. We found no difference in the mean age between the two groups; the average age of the two groups was 53.96 and 55.41, respectively (*P* = 0.221). Basic surgical methods were applied, including total or radical hysterectomy (TH), bilateral salpingo-oophorectomy (BSO), and selective pelvic or/and para-aortic lymphadenectomy. Intraoperative frozen section analysis indicated omentectomy in patients with ovarian lesions. Appendectomy was only performed when the intraoperative frozen section analysis suggested mucinous carcinoma. Before the operation began, peritoneal lavage was performed in each patient for a cytological examination. The primary surgical treatments included TH with BSO and pelvic lymphadenectomy, followed by omentectomy and pelvic/para-aortic lymph node dissection after the peritoneal cytology analysis. The data regarding the age at onset, BMI, serum CA-125 level, menopausal status, and main complaints were similar between the SCEO and ECOM groups, but the endometrial carcinoma stage, endometrial tumour grade, degree of myometrial infiltration, presence of LVSI, and five-year overall survival (OS) significantly differed between the groups (*P* < 0.001). All patients received follow-up treatment, chemotherapy or concurrent chemoradiotherapy, which are very important. Kaplan-Meier survival analysis is shown in Fig. 1. Moreover, we further classified the histological types of SCEO and ECOM(Table 2). In SCEO group, endometrial carcinoma was the main pathological type, while serous carcinoma was the main pathological type of ovarian cancer. In ECOM group, endometrial carcinoma was the primary histological type of endometrial and ovarian cancer.

**Table 1:** Clinical and pathological data for SCEO and ECOM patients

	SCEO (*N* = 51)	ECOM (*N* = 60)	*P*
MEAN AGE (Y)	53.96	55.41	0.221
BMI (KG/M2)	29.31	28.93	0.245
MENOPAUSAL STATUS (PRE, POST)	Pre: *n* = 26 (51)	Pre: *n* = 21 (35)	0.447
	Post: *n* = 25 (49)	Post: *n* = 39 (65)	
SERUM CA-125 LEVEL (U/ML)	< 35: *n* = 28 (55)	< 35: *n* = 29 (48)	0.87
	> 35: *n* = 23 (45)	> 35: *n* = 31 (52)	
	Irregular vaginal bleeding *n* = 24 (47)	Irregular vaginal bleeding *n* = 37 (62)	0.827
MAIN COMPLAINTS	Lower abdominal mass *n* = 19 (37)	Lower abdominal mass *n* = 14 (23)	
	Abdominal distention *n* = 8 (16)	Abdominal distention *n* = 9 (15)	
FIGO STAGE, ENDOMETRIAL CANCER	I: *n* = 45 (88)	I: *n* = 0 (0)	0.001
	II: *n* = 5 (10)	II: *n* = 0 (0)	
	III: *n* = 1 (2)	III: *n* = 42 (70)	
	IV: *n* = 0 (0)	IV: *n* = 18 (30)	
FIGO STAGE, OVARIAN CANCER	I: *n* = 32 (63)	NA	&&&
	II: *n* = 5 (10)	NA	
	III: *n* = 14 (27)	NA	
	IV: *n* = 0 (0)	NA	
SURGERY TYPE	TH + BSO: *n* = 2 (4)	TH + BSO: *n* = 3 (5)	0.000
	TH + BSO + PLA: *n* = 9 (18)	TH + BSO + PLA: *n* = 8 (13)	
	TH + BSO + PLA + OM: *n* = 10 (20)	TH + BSO + PLA + OM: *n* = 17 (28)	
	TH + BSO + OM: *n* = 4 (8)	TH + BSO + OM: *n* = 6 (10)	
	TH + BSO + PLA + OM + PALA: *n* = 10 (20)	TH + BSO + PLA + OM + PALA: *n* = 15 (25)	
	TH + BSO + PLA + OM + PALA+ 6: *n* = 4 (8)	TH + BSO + PLA + OM + PALA+ 6: *n* = 6 (10)	
	TH + BSO + PLA + OM + 6: *n* = 8 (16)	TH + BSO + PLA + OM + 6: *n* = 3 (5)	
	TH + BSO + 6: *n* = 1 (2)	TH + BSO + 6: *n* = 0 (0)	
	TH + BSO + PLA + PALA: *n* = 3 (6)	TPH + BSO + PLA + ALA: *n* = 2 (3)	
HISTOLOGY TYPE	Endometrioid: *n* = 33 (65)	Endometrioid: n = 37 (62)	0.001
	Not Endometrioid: *n* = 18 (35)	Not Endometrioid: *n* = 23 (38)	
ENDOMETRIAL TUMOUR GRADE	I: *n* = 33 (65)	I: *n* = 15 (25)	0.001
	II-III: *n* = 18 (35)	II-III: *n* = 45 (75)	
MYOMETRIUM INFILTRATION DEPTH	< 1/2: *n* = 40 (78)	< 1/2: *n* = 22 (37)	0.001
	≥1/2: *n* = 11 (22)	≥1/2: *n* = 38 (63)	
LVSI	(−): *n* = 38 (75)	(−): *n* = 19 (32)	0.039
	(+): *n* = 13 (25)	(+): *n* = 41 (68)	
LYMPH NODE METASTASIS	(−): *n* = 40 (78)	(−): *n* = 32 (53)	0.001
	(+): *n* = 4 (8)	(+): *n* = 20 (33)	
OMENTUM METASTASIS	(−): *n* = 30 (59)	(−): *n* = 24 (40)	0.000
	(+): *n* = 6 (12)	(+): *n* = 33 (55)	
POSTOPERATIVE THERAPY	Follow-up: *n* = 0	Follow-up: *n* = 0	0.003
	Chemotherapy: *n* = 18 (35)	Chemotherapy: *n* = 29 (48)	
	Radiotherapy: *n* = 5 (10)	Radiotherapy: *n* = 2 (4)	
	Chemoradiotherapy: *n* = 28 (55)	Chemoradiotherapy: *n* = 29 (48)

**Table 2:** The histological classification of SCEO and ECOM

Histological classification	SCEO (*n* = 51)	ECOM (*n* = 60)
Endometrial cancer	Endometrial carcinoma (*n* = 33)	Endometrial adenocarcinoma (*n* = 37)
	Serous carcinoma (*n* = 15)	Serous carcinoma (*n* = 14)
	Mucinous carcinoma (*n* = 0)	Mucinous carcinoma (*n* = 4)
	Mixed carcinoma (*n* = 1)	Clear cell carcinoma (*n* = 3)
	Clear cell carcinoma (*n* = 1)	Carcinosarcoma (*n* = 2)
	Carcinosarcoma (*n* = 1)	
Ovarian cancer	Endometrial adenocarcinoma (*n* = 19)	Endometrial adenocarcinoma (*n* = 37)
	Serous carcinoma (*n* = 23)	Serous carcinoma (*n* = 14)
	Mucinous carcinoma (*n* = 3)	Mucinous carcinoma (*n* = 4)
	Mixed carcinoma (*n* = 1)	Clear cell carcinoma (*n* = 3)
	Clear cell carcinoma (*n* = 4)	Carcinosarcoma (*n* = 2)
	Carcinosarcoma (*n* = 1)	

## Discussion

SCEO is relatively rare, and the incidence rate ranges from 2 to 8.5% [4]. Distinguishing between SCEO and ECOM is very important for proper staging and management, and histopathology is widely used in the diagnosis of both SCEO and ECOM.

In recent years, high-throughput sequencing results suggested that the most SCEOs based on pathological diagnostic criteria were actually metastases from endometrial or ovarian cancer. Molecular immunohistochemical studies can be used to help identify both SCEO and ECOM; such studies include the determination of heterozygous chromosome loss, flow cytometry, PTEN/MMAC1 mutations, K-ras and p53 gene mutations, β-catenin signalling pathway activity, microsatellite instability and protein expression levels [5, 6]. However, molecular biology findings cannot be unified. In addition, recent studies have shown that in most cases of SCEO, parallel sequencing revealed clonal consistency in most cases of SCEO with pathological diagnosis [7]. Nevertheless, sequencing diagnosis had not been widely used in clinical practise, these two types of tumours can still be distinguished by histopathological criteria [8].

The incidence rate of SCEO is relatively low [9]. Most women with ovarian or endometrial cancer are postmenopausal and in their sixties or seventies. However, most studies on concomitant ovarian and endometrial cancer have shown a younger median patient age of 40–50 years. In our study, the mean patient age was 53.96 years in the SCEO group and 55.42 years in the ECOM group, and the values were not significantly different (*P* = 0.221). There were no differences between the two groups in terms of the general data. The main complaints were abnormal vaginal bleeding, a pelvic mass, abdominal distension and abdominal pain. Meanwhile, these diseases lack effective diagnostic and therapeutic criteria. Two studies have shown that patients with SCEO are significantly younger than patients with ECOM [10, 11].

Although primary surgery has been recognized as the main treatment for SCEO, whether adjuvant therapy should be administered remains controversial. Some reports [12] showed that adjuvant therapy should be given to SCEO patients to improve their survival rate. In our study, 100% of patients in the SCEO group underwent an auxiliary computed tomography (CT) examination, and the basic procedure comprised selecting the surgical method and determining the need for TH or appendectomy. Huang et al. [13] reported a higher survival rate with lymph node excision in patients with endometrial cancer, and other studies have shown that a better prognosis for SCEO than ECOM.

A Gynecologic Oncology Group (GOG) study found a 5- and 10-year survival rate of 85.9 and 80.3%, respectively, for SCEO [14]. In our current study, we found 5-year survival rates of 58.8 and 36.7% for SCEO and ECOM, respectively. Most studies comparing SCEO to primary endometrial or primary ovarian cancer have shown a better prognosis for SCEO [15, 16]. Factors that could affect survival, such as disease stage, histological grade, tumour size, and residual tumour tissue after surgery, were compared in 77 cases of double cancer, 132 cases of primary endometrial cancer, and 126 cases of primary ovarian cancer. The results showed no differences in the 5-year survival rate among the groups (71.6, 79.8, 69.3%). These authors reported that the risk of recurrence should be considered after the treatment of double cancer, and that carboplatin and paclitaxel can be used as chemotherapy or in combination with radiotherapy [17]. A study of double cancer in 1500 patients showed that the prognosis improved with a younger age (less than 55 years), earlier stage, lower stage, the premenopausal state, and lymph node dissection, while greater omentum metastasis and residual disease were not identified by the single-factor analysis [18].

One disadvantage of our study is its retrospective nature because SCEO is uncommon. Additionally, these conditions cannot be easily diagnosed by intraoperative frozen section analysis, and most patients are diagnosed after surgery. Another limitation is that the data regarding recurrence in these patients cannot be analysed. Subsequent studies should thoroughly investigate the relationship between recurrence and prognosis in these two groups. It is expected that molecular analysis will provide more diagnostic evidence for SCEO in the further.

In conclusion, the identification of SCEO and ECOM has important clinical significance. Similar to most previous studies, our work shows that the prognosis of SCEO is better than that of ECOM and that clinicopathological findings can be used to determine the prognosis. Postoperatively, more aggressive adjuvant therapy may be considered in SCEO for older patients, postmenopausal patients and/or patients with advanced endometrial tumours, omentum metastasis, and residual tumour tissue. Although it is impossible to identify SCEO and ECOM intraoperatively, lymph node dissection should be performed in every SCEO patient.

## Abbreviations

BSO: Bilateral salpingo-oophorectomy
CT: Computed tomography
ECOM: Endometrial cancer with ovarian metastasis
FIGO: the International Federation of Gynecology and Obstetrics
GOG: A Gynecologic Oncology Group
LVSI: Lymphatic vascular space invasion
NA: Not applicable
OM: Omentectomy
OS: Overall survival
PALA: Para-aortic lymphadenectomy
PLA: Pelvic lymphadenectomy
SCEO: Synchronous primary cancers of the endometrium and ovary
TH: Total hysterectomy
